# Dual Oxygen Defects in Layered La_1.2_Sr_0.8−x_Ba_x_InO_4+δ_ (x = 0.2, 0.3) Oxide-Ion Conductors: A Neutron Diffraction Study

**DOI:** 10.3390/ma12101624

**Published:** 2019-05-17

**Authors:** Loreto Troncoso, Carlos Mariño, Mauricio D. Arce, José Antonio Alonso

**Affiliations:** 1 Instituto de Ciencia de Materiales de Madrid, CSIC, Cantoblanco, 28049 Madrid, Spain; ja.alonso@icmm.csic.es; 2 Instituto de Materiales y Procesos Termomecánicos, Universidad Austral de Chile, General Lagos, 2086, 5111187 Valdivia, Chile; carlos.marino@usach.cl; 3 Departamento de Metalurgia, USACH, Ave Ecuador 3469, 9170124 Santiago, Chile; 4 CNEA-CONICET, Centro Atómico Bariloche, Av. Bustillo 9500, S. C. de Bariloche, 8400 Río Negro, Argentina; mauricio.arce@gmail.com

**Keywords:** oxygen-ion conductor, solid electrolyte, layered perovskite, oxygen interstitials, oxygen vacancies, activation energy, neutron powder diffraction

## Abstract

The title compounds exhibit a K_2_NiF_4_-type layered perovskite structure; they are based on the La_1.2_Sr_0.8_InO_4+δ_ oxide, which was found to exhibit excellent features as fast oxide-ion conductor via an interstitial oxygen mechanism. These new Ba-containing materials were designed to present a more open framework to enhance oxygen conduction. The citrate-nitrate soft-chemistry technique was used to synthesize such structural perovskite-type materials, followed by annealing in air at moderate temperatures (1150 °C). The subtleties of their crystal structures were investigated from neutron powder diffraction (NPD) data. They crystallize in the orthorhombic *Pbca* space group. Interstitial O3 oxygen atoms were identified by difference Fourier maps in the NaCl layer of the K_2_NiF_4_ structure. At variance with the parent compound, conspicuous oxygen vacancies were found at the O2-type oxygen atoms for x = 0.2, corresponding to the axial positions of the InO_6_ octahedra. The short O2–O3 distances and the absence of steric impediments suggest a dual oxygen-interstitial mechanism for oxide-ion conduction in these materials. Conductivity measurements show that the activation energy values are comparable to those typical of ionic conductors working by simple vacancy mechanisms (~1 eV). The increment of the total conductivity for x = 0.2 can be due to the mixed mechanism driving both oxygen vacancies and interstitials, which is original for these potential electrolytes for solid-oxide fuel cells.

## 1. Introduction

Fuel cells are electrochemical devices capable of transforming the chemical energy stored in a fuel directly into electricity. The direct combustion of a fuel, e.g., hydrogen, involves a direct transfer of electrons from H to the oxidant, e.g., oxygen, taking advantage of the combustion heat as the only profit of the direct chemical reaction. The efficiency is restricted to the Carnot cycle [1] in the case of direct combustion. A fuel cell takes advantage of the electron transfer between H_2_ and O_2_ molecules, which is achieved by separating the oxidation and reduction semi-reactions with an electrolyte; here the combustion heat is just a side product. There are several types of fuel cells, of which solid-oxide fuel cells (SOFC) exhibit the highest performance; they are promising for stationary applications [2,3,4]. In a SOFC, the anode, the cathode, and the electrolyte are metal oxides; therefore, the operating temperatures are subject to the oxide-ion conduction capacity of the electrolyte. Hence, high operating temperatures (typically around 1000 °C) are required to stimulate this ionic conductivity. Many inconveniences arise from these high operation temperatures, therefore a reduction is desirable to an intermediate-temperature range between 600–850 °C. The preservation of the characteristic high efficiency requires the development of novel materials with improved properties, in particular, novel electrolytes with enhanced ionic conductivity.

The design of these materials should have strict requirements such as an oxygen-ion transport with low activation energy, low electrical resistance, and proper electrochemical kinetics. The commercial and most studied ionic conductors are based on fluorite and perovskite-type structures (Zr_1−x_Y_x_O_2−δ_, Ce_1−x_GdO_2−δ_, La_1−x_Sr_x_Ga_1−y_Mg_y_O_3−δ_) [5,6,7]. All of them exhibit oxygen ion conductivity values of σ ≥ 0.10 S/cm between 700 and 1000 °C. These exceptional conductivity values are achieved through the creation of vacancies in their framework. However, there are many examples of SOFC with alternative electrolytes working successfully below 800 °C [8,9,10,11,12,13,14].

Various alternative frameworks have been described, such as apatites [15] or garnets with La_x_Y_3−x_Fe_5_O_12+δ_ composition, where the oxide-ion conduction is based on an excess oxide-ion concentration mechanism [16].

Recently, rare-earth perovskites with K_2_NiF_4_-type structure have been described to exhibit ionic conduction. These layered structures contain an open interlayer space where an appreciable number of interstitial oxygen atoms can be hosted; therefore, they seem to be a suitable alternative to be used as electrolytes in SOFCs [17,18,19,20]. However, the conductivity of these materials is still well below the limits for them to be used as electrolytes in an SOFC system, i.e., below 0.10 S/cm at 700–1000 °C.

The atomic arrangement A_2_BO_4_ is constituted by single layers of perovskite consisting of corner-sharing BO_6_ octahedra, which alternate with a NaCl-type layer of AO atoms. There are two types of oxygens; the equatorial O1 oxygen atoms link the BO_6_ octahedra in the basal plane, while the axial O2 oxygen atoms connect the perovskite layers with the AO layer. The interstitial atoms, responsible for the oxide-ions conduction, are found in the sodium chloride type layer, only coordinated by the A cations [21]. In previous studies we described La_1+x_Sr_1−x_InO_4+δ_ (x = 0.1, 0.2) and SrIn_1−x_B_x_O_4+δ_ (B = Zr, Ti) oxides as possible solid electrolytes for SOFCs [22,23]. The best electrolyte proved to be La_1.2_Sr_0.8_InO_4.11_(1), exhibiting extremely low activation energy of only 0.51 eV for the conduction mechanism via interstitials at low temperatures (T< 650 °C), significantly smaller than those of other electrolytes working with a vacancy mechanism, typically of 1 eV.

In these layered K_2_NiF_4_-type materials, the strategy to induce the accommodation of oxygen interstitials in the NaCl layers is the creation of a positive charge unbalance at La, Sr positions; the mentioned La_1.2_Sr_0.8_ stoichiometry implies the nominal incorporation of 0.1 oxygen atoms in the interlayer space, assuming trivalent La and In and divalent Sr ions.

Based on these results, in the present work we describe the synthesis and characterization of a new family of oxides with composition La_1.2_Sr_0.8−x_Ba_0.2_InO_4±δ_ (x = 0.2, 0.3), where the introduction of Ba instead of Sr ions aims to expand the NaCl-type layer of the structure in which the ionic conduction takes place. Through a neutron powder diffraction (NPD) study we unveiled a dual defect mechanism for conduction, since both oxygen vacancies concerning the axial octahedral positions, as well as interstitial oxygen atoms where found in the interlayer space.

## 2. Experimental Section

### 2.1. Synthesis

La_1.2_Sr_0.8−x_Ba_x_InO_4+δ_ (x = 0.2, 0.3, 0.4) oxides were prepared via a citrate-nitrate route. Stoichiometric amounts of analytical grade Sr(NO_3_)_2_ (99 at.%, Strem Chemicals), La(NO_3_)_3_·6H_2_O (99.9 at.%, Alfa Aesar), Ba(NO_3_)_2_ (99 at.% Merck), and In(NO_3_)_3_·9H_2_O (99.99 at.% Alfa Aesar) were dissolved under stirring in 250 mL of 10% citric-acid (Panreac) aqueous solution with several droplets of concentrated HNO_3_ (J.T. Baker) on a porcelain capsule. This mixture was slowly evaporated on a hot plate with a magnetic stirrer at 300 °C, leading to organic resins where a random distribution of the involved cations was obtained. The resins were dried at 120 °C in a laboratory stove (Heraeus) and slowly decomposed on a muffle at temperatures up to 600 °C for 12 h (JH Hornos). Subsequent treatment at 800 °C for 2 h ensured the total elimination of all the organic materials and nitrates. Final heating at 1150 °C for 12 h gave rise to well-crystallized, homogeneous samples. 

### 2.2. Structural Characterization

The identification and characterization of the final products were carried out by X-ray diffraction (XRD) for phase identification and to assess phase purity using a Bruker-axs D8 diffractometer (40 kV, 30 mA), controlled by DIFFRACTplus software, in the Bragg–Brentano reflection geometry with CuK_α_ radiation (λ = 1.5418 Å). Neutron powder diffraction (NPD) data were collected in the High-Resolution Powder Diffractometer for Thermal Neutrons (HRPT) at the Swiss Spallation Neutron Source in the Paul Scherrer Institute (SINQ-PSI) Switzerland with a neutron wavelength λ = 1.494 Å. About 2 g of the sample was contained in a vanadium can. The measurements were carried out at 25 °C (RT). The counting time for each pattern was 3 h. The crystal structures were analyzed by the Rietveld method [24], using the FULLPROF refinement program [25]. The peak profiles were modeled by a pseudo-Voigt function. The following parameters were refined in the final runs: scale factor, background coefficients, zero-point error, pseudo-Voigt corrected for asymmetry parameters, unit-cell parameters, and positional, isotropic thermal factors for the metals and anisotropic for O1 and O2 oxygen atoms. Occupancy factors for oxygen atoms were also refined for NPD data. The coherent scattering lengths for La, Sr, In, Ba, and O were 8.240, 7.020, 4.065, 5.070, and 5.803 fermi, respectively. 

### 2.3. Conductivity Measurements

Solid bars (0.08 × 0.3 × 0.8 cm) were subjected to 2-probe DC conductivity measurements using Pt ink (PSI supplies) as the current collector. Conductivity values were acquired with an Agilent 34972A Data Acquisition Unit at a temperature range between room temperature and 1000 °C under atmospheric air [26].

## 3. Results and Discussion

### 3.1. Crystallographic Characterization

**Figure 1:**
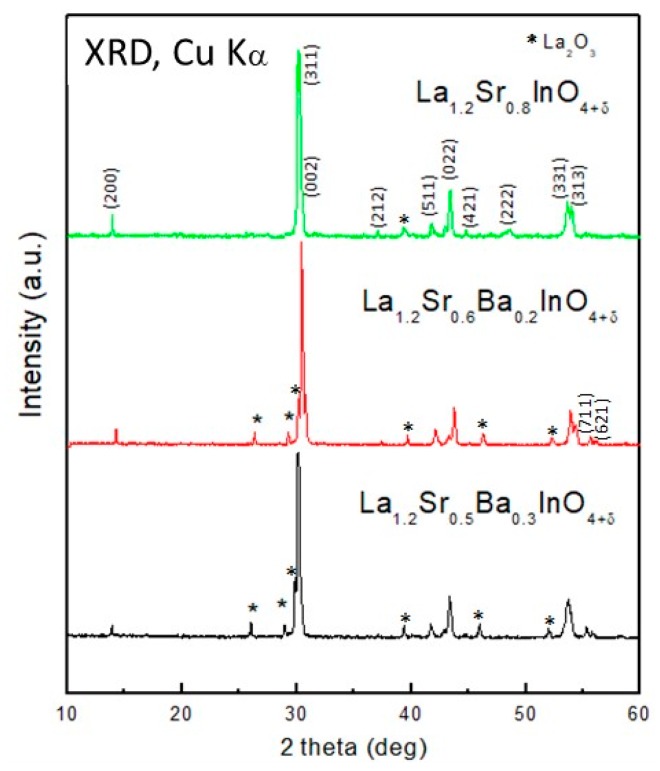
XRD patterns of the materials of the series La_1.2_Sr_0.8−x_Ba_x_InO_4+δ_, collected with CuK_α_ radiation. The stars correspond to La_2_O_3_ impurity phase.

The samples were obtained as polycrystalline, yellowish powders. Figure 1 illustrates the laboratory XRD diagrams at room temperature for the three members of the La_1.2_Sr_0.8−x_Ba_x_InO_4_ series. The patterns correspond to layered perovskites that can be indexed in the orthorhombic *Pbca* space group (Card 04-017-3962 ICDD). A small amount of La_2_O_3_ was found (Card 01-073-2141 ICSD) in all patterns.

A neutron diffraction study was essential to reveal the structural features of the three materials. The crystal structures can be defined in the *Pbca* structural model, as proposed by Titov et al. [27]. The A-type metal atoms, La, Sr, and Ba, are statistically distributed over 8c (x,y,z) positions; In atoms occupy the 4b (1/2,0,0) sites and the two types of oxygen atoms O1 and O2 are placed at two distinct 8c Wyckoff sites. The initial refinement of the unit-cell parameters (Table 1) demonstrated a monotonous increase of the unit-cell volume for x = 0.2 (435.23(5) Å^3^) and 0.3 (437.15(7) Å^3^) with respect to the parent La_1.2_Sr_0.8_InO_4_ (432.42(4) Å^3^, [23]), as it corresponds to the larger size of Ba^2+^ vs. Sr^2+^.

**Figure 2:**
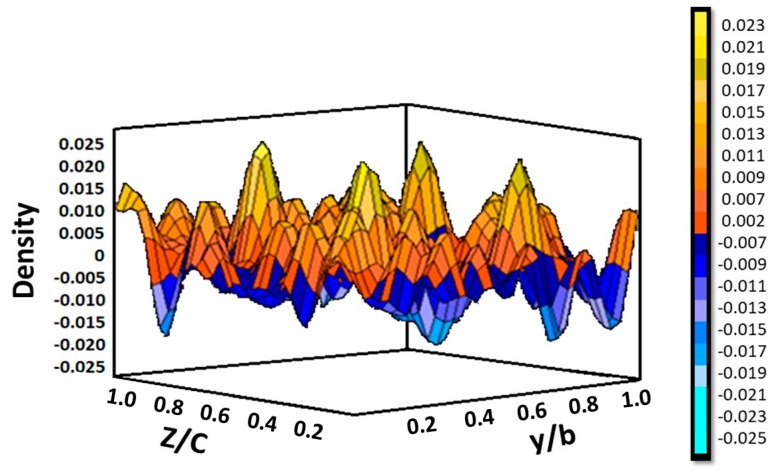
Localization by difference Fourier analysis of the interstitial oxygen atoms (O3) at 8*c* (x,y,z) sites, corresponding to the x = 0.22 layer, for La_1.2_Sr_0.6_Ba_0.2_InO_4+δ_ from neutron powder diffraction (NPD) data at room temperature.

However, a further increase in the amount of Ba to x= 0.4 does not lead to a significant increment of the unit-cell volume, which indicates that the layered perovskites cannot be further enriched with this element. In the following, only the x = 0.2 and 0.3 materials will be considered. For them, the refinement of the occupancy factor of O1 atoms yielded full stoichiometry whereas O2 displayed the presence of vacancies at the octahedral lattice; O2 are the axial oxygen atoms of the InO_6_ octahedra. Additionally, difference Fourier maps allowed the localization of extra interstitial oxygen atoms at 8c (x,y,z) sites, x ≈ y ≈ z ≈ 0.25, as positive peaks (Figure 2 for x = 0.2) placed at the NaCl layers.

**Figure 3:**
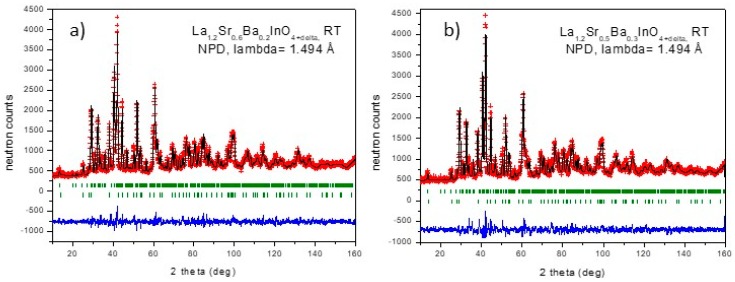
Observed (crosses), calculated (full line) and difference (at the bottom) NPD profiles for (**a**) La_1.2_Sr_0.6_Ba_0.2_InO_4+δ_, and (**b**) La_1.2_Sr_0.5_Ba_0.3_InO_4+δ_, at 25 °C, refined in the *Pbca* space group. The vertical markers correspond to the allowed Bragg reflections for the main phase; the second series of markers corresponds to La_2_O_3_ minor impurity phase.

Once introduced in the structural model as O3 atoms, a significant decrease of the R_Bragg_ discrepancy factors from 7.5% to values below 6% was observed. The combination of oxygen vacancies on O2 sites and interstitial O3 atoms yields a global oxygen stoichiometry of 4.10(2), as expected from the metal charge misbalance between La^3+^, Sr^2+^ and Ba^2+^. A minor impurity phase of La_2_O_3_ was detected in all the patterns and included in the refinement as a second phase. Figure 3a,b illustrates the quality of the Rietveld fits for La_1.2_Sr_0.6_Ba_0.2_InO_4.10(2)_ and La_1.2_Sr_0.5_Ba_0.3_InO_4.10(2)_. Table 2 contains the main atomic parameters after the refinement; Table 3 lists the anisotropic displacement parameters for O2 and O3 and Table 4 the main interatomic distances and bond angles.

**Figure 4:**
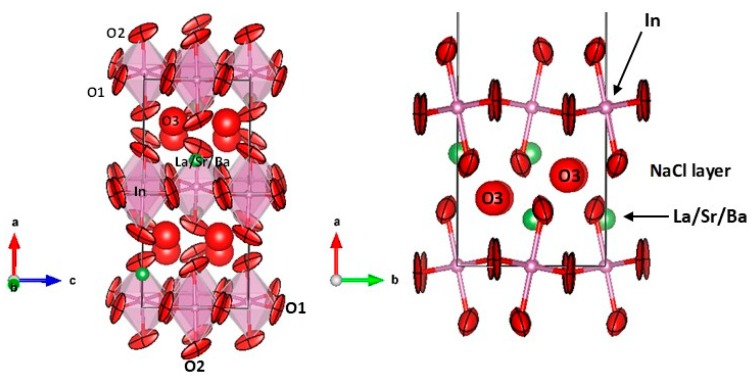
Crystal structure views of La_1.2_Sr_0.5_Ba_0.3_InO_4+δ_, showing sheets of tilted InO_6_ octahedra alternating with (La/Sr/Ba)–O layers, where interstitial O3 oxygen atoms are located, highlighting the anisotropic displacement parameters, of oblate type for equatorial O1 and prolate type for axial O2 oxygen atoms. The ellipsoids are represented with 95% of probability. The right panel highlights the NaCl layer where O3 interstitials are accommodated taking advantage of the space left by the octahedral tilting. Figures were made using the VESTA program.

Figure 4 shows two views of the orthorhombic crystal structure for x = 0.3 at RT, consisting of layers of rotated InO_6_ octahedra alternating with (La/Sr/Ba)–O layers with NaCl structure.

The InO_6_ octahedra are significantly tilted by θ = 9.16° and 7.83° for x = 0.2 and 0.3, respectively (Table 4), obtained as θ = [180 − (In–Ô–In)]/2. The reduction of the tilting angle is expected for a structure with a higher tolerance factor determined by the larger ionic size of Ba^2+^ with respect to La^3+^ or Sr^2+^. The interstitial O3 atoms occupying the NaCl layer are bonded to (La, Sr) with reasonable distances of 2.50 Å (in average) range.

In this respect, a last issue concerns the mechanism that can be induced from the structural features determined by neutron diffraction for both perovskite oxides. In principle, it is believed that the ionic transport required for the performance of the electrolyte materials for SOFC works with a vacancy mechanism, the pre-existing vacancies being filled by neighboring oxygen atoms upon conduction of oxide ions. However, in this case, the neutron data consistently indicate, for both compounds at RT, that the layered perovskites contain both oxygen vacancies located at the axial oxygen atoms of the InO_6_ octahedra as well as interstitial oxygens conspicuously located in the NaCl layers. As it is plausible that all these materials combine an excellent ionic conductivity with a sufficient ionic transport of oxide anions, as demonstrated for the parent La_1+x_Sr_1−x_InO_4+δ_ materials [23], we could imagine a mixed mechanism where the existing vacancies at O2 are filled with O3 atoms and with no steric impediment, giving rise to a fast transport of oxide ions across the NaCl layers. The large values of the displacement parameters (Table 3) for O3 and the anisotropic, cigar-shaped anisotropic displacement parameters (ADPs) for axial O2 oxygens are a fixed picture of a dynamic situation involving very reactive atoms, the lability being induced by the presence of very basic Ba^2+^ ions prone to form weak covalent bonds to oxygen. 

### 3.2. Electrical Conductivity Measurements

The total conductivity was measured to compare our samples with the parent La_1.2_Sr_0.8_InO_4.11_ [23]. The results show a classical behavior of ionic conductivity in ceramic materials: It is a thermally activated or Arrhenius-type process given by Equation (1), where σ is the conductivity in (S/cm), T is the absolute temperature in (K), A is a pre-exponential factor, Ea is the activation energy in (J) and k_B_ is the Boltzmann constant in (J/K).

(1) σ(T)=Ae−EakBT

**Figure 5:**
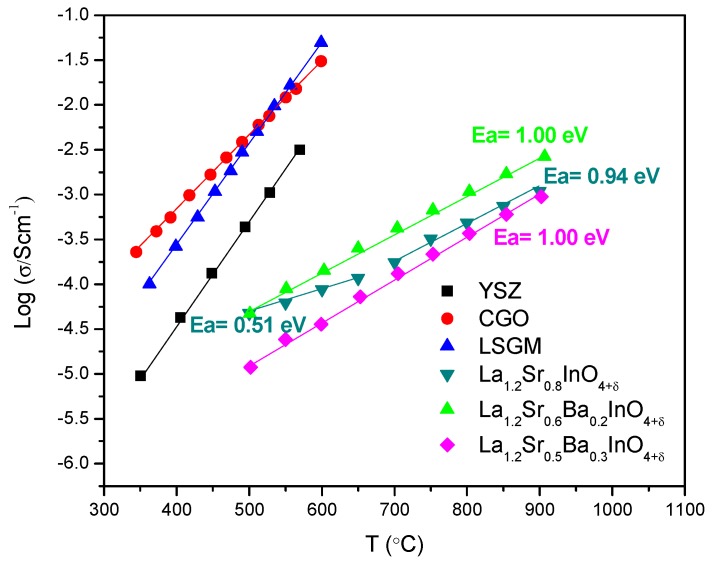
Conductivity for different electrolytes compared to La_1.2_Sr_0.8−x_Ba_x_InO_4+d_ Reference [23].

Figure 5 shows the graph of the thermal variation of total conductivity (σ) in air for La_1.2_Sr_0.8−x_Ba_x_InO_4+δ_ (x = 0.0, 0.1, 0.2) compared with that of commercial electrolytes. The parent sample (x = 0.0), as reported in Reference [23], shows an unusually low activation energy (0.51 eV) in the range between 500 and 700 °C, where a change of slope occurs, making it competitive with the electrolytes of La_1−x_Sr_x_Ga_1−y_Mg_y_O_3−δ_, Ce_1−x_GdO_2−δ_ and Zr_1−x_Y_x_O_2−δ_, at low temperatures. In that case [23], NDP data proved no oxygen vacancies inside the structure. The Ba-doped oxides present values of Ea = 1.00 eV, for both x = 0.2 and 0.3. The activation energy values are similar to those typically exhibited by ionic conductors working by a vacancy mechanism (~1 eV), but as shown in Figure 5, they are still several orders of magnitude lower in conductivity than commercial electrolytes. Although, in the case of these barium-doped samples, there is no evidence of changes in the activation energy as in the parent compound, the sample La_1.2_Sr_0.6_Ba_0.2_InO_4.10_ increases by 1 order of magnitude its conductivity, and at low temperatures, the tendency to be competitive with the typical electrolytes remains. In Table 2, it is observed that the occupation of the interstitial oxygens (O3) doubles the amount observed in the parent compound, due to the extra space provided by the insertion of the Ba cation, and at the same time, it presents an appreciable amount of vacancies at O2 sites. The increase in conductivity can be a result of the mixed mechanism driven by the presence of both oxygen vacancies and interstitials. 

In other words, the layered perovskite of La_1.2_Sr_0.6_Ba_0.2_InO_4.10_ is among the few oxides containing a measurable concentration of permanent vacancies as well as adjacent interstitial atoms in the interlayer space, which together can participate in the conduction mechanism to ensure a fast ionic transport.

## 4. Conclusions

La_1+x_Sr_1−x_InO_4+δ_ layered perovskites have recently been described as suitable candidates for electrolyte materials in SOFCs [22,23]. Based on the material with the best properties, La_1.2_Sr_0.8_InO_4+δ_, which contains a substantial amount of interstitial oxygen atoms that easily move in the interlayer plane, here we designed a unique series containing Ba^2+^ ions. Its larger ionic size drives the expansion of the unit cell, where a better oxygen conduction is expected. A neutron diffraction study reveals novel and unexpected features, since oxygen vacancies at the axial O2 octahedral positions were detected, besides the interstitial oxygen atoms located in the NaCl-type layer. Difference Fourier maps from NPD data at RT clearly show prominent positive peaks corresponding to the O3 interstitial oxygen atoms; the short distances with the oxygen vacancies make possible a dual jump mechanism where both point defects participate. Moreover, the strongly anisotropic, cigar-shaped ellipsoids for O2 mimic the displacement of axial oxygen atoms in the interlayer space, also anticipating an excellent ionic motion for oxide ions. The low activation energies measured for x = 0.2 and x = 0.3 compounds endorses this hypothesis. The presence of Ba ions in this novel series, besides the forecasted expansion of the framework, induces a second beneficial effect: the basic character of these cations, more prone to form weaker chemical bonds, favor the formation of oxygen vacancies in a crystal structure involving very labile atoms where oxygen conduction is promoted in the (La,Sr,Ba)–O (NaCl-type) layers.

## Figures and Tables

**Table 1:** Unit-cell parameters and volume at room temperature for La_1.2_Sr_0.8–x_Ba_x_InO_4_.

Reticular Parameters	0.0 *	0.2	0.3	0.4
*a* (Å)	12.6085(6)	12.6267(9)	12.640(1)	12.635(2)
*b* (Å)	5.8789(3)	5.9000(4)	5.9073(5)	5.9016(8)
*c* (Å)	5.8338(3)	5.8421(4)	5.8546(6)	5.863(1)
*V* (Å^3^)	432.42(4)	435.23(5)	437.15(7)	437.22(11)

* From Reference [23].

**Table 2:** Structural parameters and discrepancy factors after the Rietveld refinement of La_1.2_Sr_0.6_Ba_0.2_InO_4+δ_ in the space group *Pbca* from NPD data.

Atoms		x = 0.0 *	x = 0.2	x = 0.3
La/Sr/Ba 8c (x,y,z)	x	0.1458(8)	0.1469(8)	0.1463(1)
	y	−0.0155(2)	−0.0146(3)	−0.0124(4)
	z	0.9726(2)	0.9928(6)	1.0019(9)
	*B*(Å^2^)	1.14(5)	1.52(1)	1.51(7)
	*focc*	0.59(1)/0.41(1)	0.6/0.3/0.1	0.6/0.25/0.15
In 4b (1/2 0 0)	*B*(Å^2^)	0.69(11)	0.60(1)	0.68(13)
	*focc*	1.0	1.0	1.0
O1 8c (x,y,z)	x	0.0269(1)	0.0219(2)	0.0199(3)
	y	0.2161(3)	0.2322(5)	0.2407(1)
	z	0.2120(5)	0.2224(4)	0.2267(6)
	*focc*	1.0	1.0	1.0
O2 8c (x,y,z)	x	0.3268(1)	0.3236(2)	0.3251(3)
	y	0.0807(3)	0.0752(4)	0.0672(6)
	z	0.0301(4)	0.0146(9)	−0.0142(5)
	*focc*	1.0	0.897(2)	0.906(3)
O3 8c (x,y,z)	x	0.216(2)	0.2135(8)	0.2009(2)
	y	0.249(5)	0.2393(4)	0.2217(1)
	z	0.267(5)	0.1901(4)	0.2196(9)
	*B*(Å^2^)	3.88(1)	3.4(3)	6.6(1)
	*focc*	0.054(6)	0.103(2)	0.144(3)
Discrepancy Factors	χ^2^	2.80	2.36	2.72
	R_p_ (%)	4.44	4.39	4.38
	R_wp_ (%)	5.96	5.61	5.71
	R_Bragg_ (%)	5.98	5.97	6.93

* From Reference [23].

**Table 3:** Anisotropic displacement factors for O1 and O2 in La_1.2_Sr_0.6_Ba_0.2_InO_4+δ_ from NPD data.

Atom	x	0.0 *	0.2	0.3
O1 8c (x,y,z)	β_11_	26(3)	71(5)	109(8)
	β_22_	61(10)	14(11)	81(16)
	β_33_	126(13)	111(14)	122(19)
	β_12_	−27(5)	5(9)	16(22)
	β_13_	5(5)	−5(8)	72(17)
	β_23_	44(10)	30(0)	70(0)
O2 8c (x,y,z)	β_11_	16(2)	35(3)	49(5)
	β_22_	57(9)	79(11)	129(18)
	β_33_	262(15)	279(22)	478(42)
	β_12_	13(4)	17(5)	−14(9)
	β_13_	16(6)	−36(15)	−85(22)
	β_23_	20(11)	−44(22)	175(37)

* From Reference [23].

**Table 4:** Main interatomic distances (Å) and angles (°) for La_1+x_Sr_1−x_In_4+δ_ (x = 0.0, 0.2, and 0.3) determined from NPD data at RT.

x	0.0 *	0.2	0.3
La/Sr/Ba–O1	2.468(2)	2.532(3)	2.553(6)
La/Sr/Ba–O1	2.695(2)	3.089(3)	3.011(6)
La/Sr/Ba–O1	2.767(2)	2.788(3)	2.832(5)
La/Sr/Ba–O1		2.786(4)	2.779(6)
La/Sr/Ba–O2	2.366(2)	2.297(3)	2.311(4)
La/Sr/Ba–O2	2.636(3)	2.841(6)	3.060(10)
La/Sr/Ba–O2	2.425(2)	3.092(6)	2.874(10)
La/Sr/Ba–O2		2.452(3)	2.511(4)
La/Sr/Ba–O3	2.621(5)	2.067(12)	2.01(5)
La/Sr/Ba–O3	2.694(4)	2.827(11)	2.83(4)
La/Sr/Ba–O3	2.81(2)	2.559(11)	2.80(4)
La/Sr/Ba–O3	2.15(2)	2.546(12)	2.48(5)
In–O1 (x2)	2.115(2)	2.064(3)	2.042(5)
In–O1 (x2)	2.122(2)	2.141(3)	2.155(5)
In–O2 (x2)	2.249(2)	2.273(3)	2.248(4)
In–O1–In	155.55(8)	161.68(10)	164.34(19)

* From Reference [23].
